# Caregivers' Medical Care Provision at Patient End-of-Life: Associations with Emotional Difficulty and Gains

**DOI:** 10.1093/geroni/igab046.2977

**Published:** 2021-12-17

**Authors:** Amanda Leggett, Hyun Jung Koo, Elaina Baker, Hannah Lee

**Affiliations:** 1 The University of Michigan, Ypsilanti, Michigan, United States; 2 University of Michigan, Ann Arbor, Michigan, United States

## Abstract

Family caregivers play crucial roles in patient care and medical decision making, especially at end-of-life (EOL). Yet, most research focuses on caregivers’ burden, with little attention to rewards that make for a fulfilling EOL care experience. We consider caregiver involvement at EOL and associations with caregiver stress and gains. Data are drawn from the 2017 National Study of Caregiving’s last month of life and core interviews which includes caregivers (n=283) for a nationally representative sample of Medicare eligible older adults, and questions caregivers about their care provision and EOL experience. We consider indicators of caregivers’ involvement in medical decision making and support received from providers as predictors of caregivers’ emotional difficulty and gains at EOL utilizing linear regressions controlling for demographic characteristics. Caregivers were 60.7 years of age on average, 72.5% female, 21.3% non-white, and 11% were spousal partners. Making medical decisions was associated with increased emotional difficulty at EOL (B=0.93, SE=0.24, p<.001). In contrast, more caregiving gains were associated with having care decisions align with the CG’s wishes (B=-0.64, SE=0.30, p<.05), being more informed by providers (B=0.41, SE=0.16, p<.05), helping the care recipient with anxiety or sadness (B=0.69, SE=0.28, p<. 05), and surprisingly, feeling that care decisions were made without their input (B=0.82, SE=0.29, p<.01). Being more involved and informed in care was associated with both positive and negative caregiver outcomes at EOL. Understanding caregiver emotional difficulty and gains at EOL are critical for identifying how clinicians can better support caregivers at EOL and improving the caregiving experience.

